# Effects of Multivitamin-Mineral Supplementation on Chronic Stress-Induced Oxidative Damage in Swiss Albino Mice

**DOI:** 10.7759/cureus.61896

**Published:** 2024-06-07

**Authors:** Nida Suhail, Tehreem Aftab, Anwar Alruwaili, Daliyah Alruwaili

**Affiliations:** 1 Department of Medical Laboratory Technology, Faculty of Applied Medical Sciences, Northern Border University, Arar, SAU; 2 Department of Physiology, Faculty of Medicine, Northern Border University, Arar, SAU

**Keywords:** swiss albino mice, unpredictable chronic stress, oxidative damage, multivitamin-mineral, antioxidants

## Abstract

Objective: Stress is a hazardous occurrence that causes a variety of physiological and behavioral responses in a person. It increases energy metabolism and enhances oxidative stress, both of which are implicated in the pathophysiology of several diseases. Numerous vitamins and minerals have the ability to modulate oxidative stress. The present investigation aimed to evaluate the effectiveness of a multivitamin-mineral (MM) supplement in addressing oxidative imbalances caused by chronic stress in the plasma, hepatic, and renal tissues of Swiss albino mice.

Methods: Thirty healthy male Swiss albino mice were randomly assigned to one of the three groups, with 10 animals each: control, unpredictable chronic stress (UCS), and MM + UCS. The experiment lasted for four weeks, after which all the animals underwent cervical decapitation, and samples of their blood, liver, and kidney were taken for biochemical studies. DNA damage analysis was performed on lymphocytes.

Results: Exposure to UCS negatively affected all biochemical markers, as indicated by reduced levels of antioxidants (superoxide dismutase, catalase, glutathione S-transferase, glutathione reductase, and reduced glutathione) in the plasma, liver, and kidney tissues, along with enhanced levels of lipid peroxidation and marker enzymes. MM supplementation normalized the deranged biochemical markers in stress-exposed mice. The results of DNA damage supported the biochemical findings mentioned above.

Conclusion: The findings suggest that MM supplementation could help reduce oxidative disturbances caused by stress in both healthy and diseased conditions.

## Introduction

Stress is a hazardous occurrence that causes a variety of physiological and behavioral responses in a person. It increases energy metabolism and enhances oxidative stress, both of which are implicated in the pathophysiology of numerous diseases [1]. Stressful circumstances are among the most prevalent experiences that people encounter. Within a theoretical framework pertaining to stress and illness, biological and behavioral factors are recognized to impact various facets involved in tumor development, such as immune escape to the metastatic cascade, angiogenesis, invasion, and apoptosis [2]. Stress-induced activation of several pathways can enhance the formation of reactive oxygen species (ROS) [3]. Despite the effective defenses provided by DNA repair enzymes and apoptotic pathways, reactive oxygen and nitrogen species under oxidative stress induce strand breaks, crosslinks, and a host of other alterations that result in mutations [4,5].

These days, multivitamin-mineral (MM) supplements are becoming more popular as a preventative and disease-mitigation measure. Numerous micronutrients, including antioxidant vitamins and minerals, have the ability to modulate oxidative stress [6]. MM supplements are an important means of micronutrient consumption and are favored by a lot of people for prophylaxis [7]. They provide reasonable insurance against diseases such as heart disease, depression, cataracts, immunological function, and cancer, especially for individuals who have poor diets. Telomere length, a biological aging marker, was found to be increased in women who took multivitamin supplements on a regular basis [8]. In healthy children, MM enhances cognitive and mood benefits [9]. MM supplementation has also been proven to be inversely linked to the risk of myocardial infarction in both genders [10]. There is, however, a dearth of information on effectiveness trials assessing the functions of MM supplements in mitigating stress-induced oxidative damage.

The present investigation aimed to evaluate the effectiveness of an MM supplement in addressing oxidative imbalances caused by chronic stress in the plasma, hepatic, and renal tissues of Swiss albino mice.

## Materials and methods

Chemicals

MM supplement (tablet zincovit) was acquired from Apex Laboratories Pvt. Ltd. India. The following are the compositions of the MM tablet: vitamin A 5000 IU, vitamin D3 400 IU, vitamin E 15 mg, vitamin B12 7.5 mcg, vitamin B2 10 mg, vitamin B6 2 mg, vitamin C 75 mg, vitamin B5 10 mg, vitamin B1 10 mg, niacinamide 50 mg, magnesium 18 mg, copper 0.5 mg, manganese 0.9 mg, zinc 22 mg, selenium 50 mcg, folic acid 1 mg, biotin 150 mcg, iodine 150 mcg, chromium 25 mcg, molybdenum 25 mcg, carbohydrate 0.2 g.

DMBA, Histopaque 1077, HBSS, and RPMI 1640 were obtained from Sigma Aldrich (St. Louis, MO). All the other chemicals utilized in the study were of analytical grade and purchased from Sisco Research Laboratories (SRL), Qualigens, and Span Diagnostics Ltd., India.

Experimental design

The study was conducted at AM University, Aligarh, India. Prior to the start of the treatment, 30 healthy male Swiss albino mice (weight: 40-45 g, age: 5-6 weeks) were acclimatized for a one-week to 12-hour light/dark cycle and a standard mice feed (carbohydrate: 48.8%, protein: 21%, fat: 3%, fiber: 5%, ash: 8%, calcium: 0.8%, phosphorus: 0.4%, moisture: 13%) with free access to water. The Institutional Animal Ethics Committee approved the study, and all procedures followed the guidelines set forth by the Committee for Control and Supervision of Experiments on Animals (714/02/a/CCSEA).

Mice were randomly allocated into three groups of 10 each, as shown in Table 1. The unpredictable chronic stress (UCS) procedure was performed according to the method of Oritz et al. [11]. The following are the details of the UCS procedure: to implement wet bedding, 300 milliliters of tap water were added to the home cage. Mice were kept individually in body-sized wire mesh cages fastened to wooden planks for the purpose of restraint stress, with no access to movement. Crowding was done by putting an iron barrier in the cage to provide the smallest amount of living space possible. In order to perform a forced swim and a cold forced swim, the mice were placed in a cylindrical tank (50 cm in height x 20 cm in diameter) filled with water to a depth of 20 cm at either 25°C or 4°C, respectively. Lastly, illumination was attained by placing an illuminated tube light on the cages overnight. The weekly protocol of UCS is outlined in Table 2. Animals were held in a recovery area for one to two hours after each stressor before being put in clean cages and transferred back to the animal house. Genetic predispositions were excluded in the current study. MM supplementations were prepared fresh on a daily basis. The dose and duration of the MM supplement (200 mg/kg body weight) for mice were based on the published research by Mani Satyam et al. [12], with slight modifications. The weight of the mice was recorded every seven days to adjust the supplement doses.

**Table 1:** Treatment protocol UCS: unpredictable chronic stress, MM: multivitamin-mineral

Number of groups	Name of the group (10 mice/group)	Treatment
I	Control	None
II	UCS	Mice were subjected to four weeks of UCS
III	MM + UCS	200 mg/kg body weight [12] of multivitamin-mineral in 100 μl of drinking water was given orally to this group on a daily basis, after which they were subjected to the same stressor as in group II

**Table 2:** Weekly protocol of UCS UCS: unpredictable chronic stress

Day	Stress type and schedule
1	1000 h, wet bedding (25ºC), 3 h
2	0900 h, restraint, 4 h
3	1200 h, crowding, 3 h
4	1400 h, forced swim (25ºC), 30 min; 1800 h, food deprivation, overnight
5	1900 h, lights on, overnight
6	1000 h, cold forced swim (4ºC), 15 min
7	1000 h, restraint, 3 h; 2100 h, crowding, overnight

After four weeks, animals from each group were subjected to cervical decapitation, and samples of their blood, liver, and kidney were taken for biochemical analysis.

The heparinized blood was centrifuged for five minutes at 3000 rpm to extract plasma. Liver and kidney samples were obtained by first washing them with normal saline to remove cellular debris and homogenizing them in 0.1 M phosphate buffer pH 7.4 (10% w/v), followed by centrifugation at 10,000 g at 4 °C for 15 minutes. Both the plasma and the clear supernatant obtained were used for further analysis.

Biochemical analysis

For antioxidant enzymes, the antioxidant enzymes superoxide dismutase (SOD), catalase (CAT), glutathione-s-transferase (GST), and glutathione reductase (GR) were assayed in the plasma, liver, and kidney tissues according to the published procedures [13-16]. Results were expressed as enzyme units per mg of protein.

For the measurement of total reduced glutathione (GSH), the GSH level in the plasma, liver, and kidney tissues was assessed using sulfosalicylic acid and 5-5-dithiobis-2-nitrobenzoic acid, in accordance with the methodology of Jollow et al. [17]. Results were represented as micromoles of GSH/mg protein.

For lipid peroxidation, lipid peroxidation in the plasma, liver, and kidney tissues was measured by spectrophotometer using a thiobarbituric acid (TBA) assay for the generation of TBARS during an acid-heating reaction [18]. The pink color produced was identified at 535 nm and measured by using an extinction coefficient of 1.56 × 105 M/cm.

For the measurement of marker enzymes glutamic oxaloacetic transaminase (GOT) and glutamic pyruvate transaminase (GPT), transaminases (GOT and GPT) were measured in plasma and liver tissues by using commercial kits (Span Diagnostics Ltd., India). For the measurement of protein content, the method of Lowry et al. was employed for estimating the protein content in plasma, liver, and kidney tissues [19].

Single-cell gel electrophoresis (comet assay) for analyzing DNA damage

The degree of DNA damage was assessed by diluting heparinized blood in PBS (Ca++ and Mg++-free), and lymphocytes were isolated using Histopaque 1077 and then suspended in RPMI 1640. A trypan blue exclusion test was used to assess the viability of cells [20]. The comet assay was carried out according to the procedure of Muqbil et al. [21]. Tail length (migration of DNA from the nucleus, µm) was used for measuring DNA damage, which was automatically produced by the Komet 5.5 image analysis system.

Statistical analysis

The data was analyzed using SPSS Statistics version 20 (IBM Corp. Released 2011. IBM SPSS Statistics for Windows, Version 20.0. Armonk, NY: IBM Corp.). The data was presented as the group mean ± SEM of 10 values and subjected to a one-way ANOVA followed by Tukey's post hoc analysis for identifying differences among the treatment and control groups. A p-value of <0.05 was considered statistically significant.

## Results

Stress exposure for four weeks significantly (p<0.001) reduced the levels of all antioxidant enzymes (SOD, CAT, GST, and GR) in plasma, kidney, and liver tissues of mice, accompanied by an increased concentration of marker enzymes GOT and GPT in plasma and liver as compared to controls. MM supplementation was observed to have a protective role against stress-induced oxidative damage, as evidenced by significantly (p<0.001) increased antioxidant enzyme activities and decreased levels of marker enzymes in the MM + UCS group as compared to the UCS group (Tables 3-5).

**Table 3:** Effect of stress and MM supplementation on the levels of antioxidant and marker enzymes in the circulation of mice Each value represents the mean ± SEM of 10 animals in each group ^a^p<0.001 when compared to the control group ^b^p<0.001 when compared to the UCS group UCS: unpredictable chronic stress, MM: multivitamin-mineral, SOD: superoxide dismutase, CAT: catalase, GST: glutathione-s-transferase, GR: glutathione reductase, SGOT: serum glutamic oxaloacetic transaminase, SGPT: serum glutamic pyruvic transaminase

Groups (10 mice/group)	SOD (U/mg protein)	CAT (U/mg protein)	GST (U/mg protein)	GR (U/mg protein × 10^-3^)	SGOT (U/ml)	SGPT (U/ml)
Control	25.18 ± 0.30	1.87 ± 0.07	1.96 ± 0.03	2.99 ± 0.06	15.02 ± 0.04	21.33 ± 0.14
UCS	18.04 ± 0.38^a^	0.92 ± 0.06^a^	0.98 ± 0.03^a^	1.69 ± 0.11^a^	19.73 ± 0.15^a^	24.29 ± 0.29^a^
MM + UCS	22.38 ± 0.41^b^	1.64 ± 0.09^b^	1.72 ± 0.05^b^	2.57 ± 0.12^b^	16.88 ± 0.19^b^	22.08 ± 0.26^b^

**Table 4:** Effect of stress and MM supplementation on the levels of antioxidant and marker enzymes in the liver of mice Each value represents the mean ± SEM of 10 animals in each group ^a^p<0.001 when compared to the control group ^b^p<0.001 when compared to the UCS group UCS: unpredictable chronic stress, MM: multivitamin-mineral, SOD: superoxide dismutase, CAT: catalase, GST: glutathione-s-transferase, GR: glutathione reductase, GOT: glutamic oxaloacetic transaminase, GPT: glutamic pyruvic transaminase

Groups (10 mice/group)	SOD (U/mg protein)	CAT (U/mg protein)	GST (U/mg protein)	GR (U/mg protein × 10^-3^)	GOT (U/ml)	GPT (U/ml)
Control	172.56 ± 0.75	97.53 ± 0.47	92.31 ± 0.62	2.12 ± 0.04	75.72 ± 0.71	77.87 ± 0.55
UCS	104.66 ± 1.92^a^	71.92 ± 0.55^a^	85.60 ± 0.89^a^	1.23 ± 0.04^a^	88.80 ± 0.47^a^	93.47 ± 0.56^a^
MM + UCS	149.22 ± 1.63^b^	89.92± 0.68^b^	89.86 ± 0.61^b^	1.85 ± 0.04^b^	81.82± 0.52^b^	84.31 ± 0.83^b^

**Table 5:** Effect of stress and MM supplementation on the levels of antioxidant enzymes in the kidney of mice Each value represents the mean ± SEM of 10 animals in each group ^a^p<0.001 when compared to the control group ^b^p<0.001 when compared to the UCS group UCS: unpredictable chronic stress, MM: multivitamin-mineral, SOD: superoxide dismutase, CAT: catalase, GST: glutathione-s-transferase, GR: glutathione reductase, GOT: glutamic oxaloacetic transaminase, GPT: glutamic pyruvic transaminase

Groups (10 mice/group)	SOD (U/mg protein)	CAT (U/mg protein)	GST (U/mg protein)	GR (U/mg protein × 10^-3^)
Control	165.98 ± 0.69	112.38 ± 0.88	38.84 ± 0.64	4.9 ± 0.07
UCS	104.40 ± 1.62^a^	82.93 ± 1.01^a^	21.02 ± 0.45^a^	2.99 ± 0.05^a^
MM + UCS	146.93 ± 1.24^b^	102.03 ± 0.79^b^	31.14 ± 0.57^b^	4.13 ± 0.04^b^

**Figure 1:**
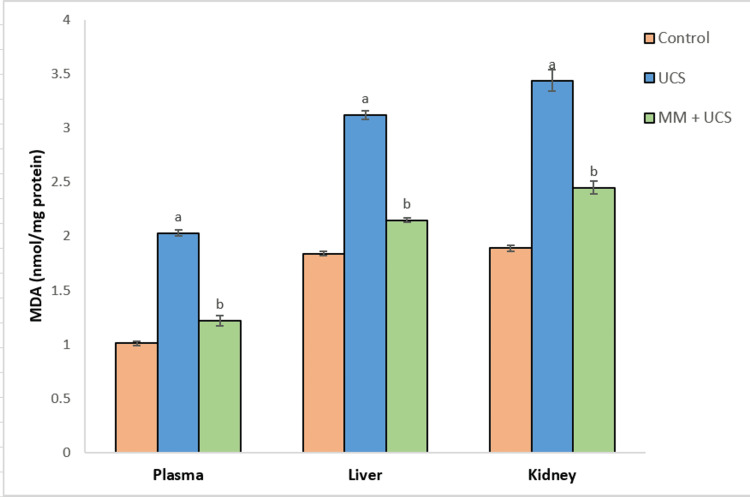
MDA levels in circulation, liver, and kidney of mice on UCS and MM + UCS treatments Data represent mean ± SEM of 10 mice in each group ^a^p<0.001 when compared to the control group ^b^p<0.001 when compared to the UCS group MDA: malondialdehyde, UCS: unpredictable chronic stress, MM: multivitamin-mineral

**Figure 2:**
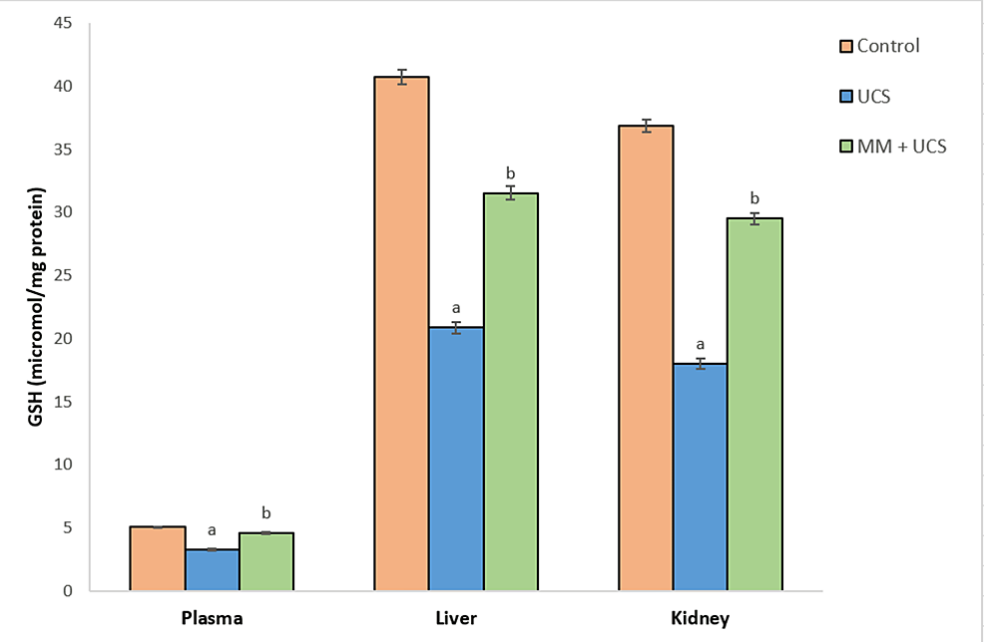
Alterations in the levels of GSH in the circulation, liver, and kidney of mice on UCS and MM + UCS treatments Data represent mean ± SEM of 10 mice in each group ^a^p<0.001 when compared to the control group ^b^p<0.001 when compared to the UCS group GSH: reduced glutathione, UCS: unpredictable chronic stress, MM: multivitamin-mineral

Exposure to stress elicited lipid peroxidation in the plasma, kidney, and liver tissues of mice, as shown by significantly elevated malondialdehyde (MDA) levels (p<0.001) in the stressed mice as compared to controls (Figure 1). However, simultaneous treatment with MM significantly reduced (p<0.001) the MDA levels compared to the stressed group. GSH levels in plasma, liver, and kidney tissues declined significantly (p<0.001) on exposure to stress, as seen in the UCS group when compared to controls. Administration of MM triggered a significant (p<0.001) increase in GSH levels as compared to the UCS group (Figure 2).

**Figure 3:**
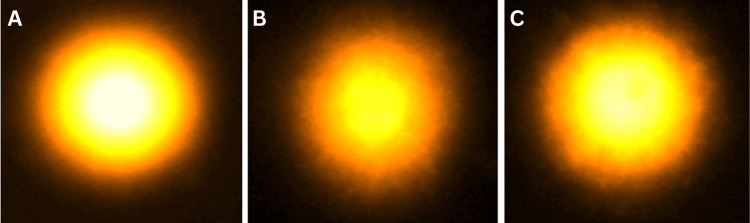
Single-cell gel electrophoresis of mice lymphocytes showing comets (100×). (A) Control, (B) UCS, and (C) MM + UCS UCS: unpredictable chronic stress, MM: multivitamin-mineral

Figure 3 illustrates the lymphocytic DNA damage caused by stress exposure. Significant damage to the DNA of lymphocytes (p<0.001) was observed in stress-exposed mice, as demonstrated by an increased tail length in the UCS group (8.93 ± 0.06 µm) as compared to controls (2.3 ± 0.06 µm). Treatment with MM (p<0.001) significantly diminished the amount of damage (4.76 ± 0.11 µm) in lymphocytes as compared to the UCS group.

## Discussion

In the current study, exposure to four weeks of UCS caused a substantial decline in the levels of all antioxidants with a concomitant increase in lipid peroxidation in the plasma, hepatic, and renal tissues of mice. Supplementation of stress-exposed mice with MM recovered their depleted antioxidant levels. In the current investigation, the UCS model (with varying stressor types and timings) was employed to imitate the chronic stressful situations that humans are frequently exposed to in their daily lives [11].

The biochemical markers in the blood and tissues, as well as the antioxidant status, were all negatively impacted by UCS. The antioxidant enzymes SOD and CAT play a crucial role in maintaining homeostasis for regular cell function and are commonly considered markers of oxidative stress. UCS exposure significantly reduced the circulatory, renal, and hepatic levels of SOD and CAT. MM administration restored the enzyme activities to near-normal levels. MM included minerals like Cu, Zn, Mn, and Fe in addition to the primary antioxidant vitamins like C, E, and β-carotene. These minerals function as co-factors for the antioxidant enzymes CAT and SOD. Furthermore, copper is required for proper iron utilization, which is an essential part of CAT. As a result, micronutrient supplementation boosted the antioxidant enzyme system.

GST, a phase II enzyme, plays a physiological role in the detoxification of harmful free radicals by directly scavenging ROS and reinstating the homeostatic redox status of normal cells [22]. A significant diminution in the levels of GST, GR, and GSH was seen upon exposure to UCS, suggesting impaired glutathione metabolism and a compromised antioxidant defense mechanism. A drop in the level of GR might be because of the deleterious effects of free radicals on the enzyme, resulting in GSH levels. However, MM was effective in detoxifying endogenous substances such as peroxidized lipids, as evidenced by the enhanced activities of phase II enzymes and GSH in the plasma and tissues of mice after MM supplementation.

Moreover, the efficacy of MM is demonstrated by reduced MDA levels, an indicator of oxidative damage that was augmented in the plasma and tissues following exposure to UCS for four weeks. The increased lipid peroxidation could be due to a considerable increase in H2O2 production and GSH depletion following stress exposure. A comparable increase in lipid peroxidation after stress exposure has been documented before in the hepatic and renal tissues of mice [23]. A decrease in the MDA levels on MM supplementation may be attributed to an alteration of the in vivo defense mechanism, which offered protection against lipid peroxidation by decomposing the peroxides. The production of increased amounts of GSH, other antioxidants, and GST may be responsible for at least some of the effective antioxidant action of MM.

Stress exposure enhanced the levels of SGOT and SGPT in plasma and liver, indicating liver dysfunction caused by hepatocyte cellular necrosis and increased membrane permeability. Treatment with MM lowered these levels, possibly by inhibiting the activation of the hypothalamic-pituitary-adrenal axis and sympathetic system in response to stress.

The increased oxidative stress also causes DNA strand breakage, resulting in oxidative modification and DNA damage [4,5], as shown by the longer tail length in the UCS group compared to the controls. Damaged DNA impairs cells' ability to repair or prevent illness. Dietary supplementation with MM was helpful in modulating oxidative stress, as indicated by reduced levels of DNA damage (decreased tail length) in the MM-supplemented group. Many vitamins and minerals are crucial for DNA replication and repair because they act as substrates or cofactors in the process [24]. Numerous studies have linked deficits in micronutrients, such as choline, iron, zinc, and biotin, to oxidant release, mitochondrial degradation, and DNA damage, including chromosome breakage, in vivo or in cultured human cells [24]. Consequently, the restored antioxidant function might have contributed to a reduction in DNA damage in the MM-treated group as compared to the UCS group.

Thus, MM conferred a considerable degree of protection to cellular macromolecules from oxidative damage by scavenging free radicals, which could have damaged their biological properties and ultimately led to cell death.

As a novel finding of the current investigation, MM exhibited strong antioxidant ability in normalizing the levels of deranged biochemical parameters. The nutrients present in MM act synergistically with the in vivo antioxidants to restore the disturbed oxidant status. Flavonoids and carotenoids serve as exceptional preventative antioxidants owing to their ability to quench singlet oxygen [25,26]. On the contrary, vitamins C and E act as effective antioxidants by neutralizing peroxyl radicals [27]. The in vivo antioxidants ceruloplasmin and glutathione peroxidase require trace elements such as Cu and Se for their activity [28,29]. Magnesium ions serve as an important cofactor for many proteins located in the intermembrane compartment of mitochondria [30]. Vitamin B6, riboflavin, biotin, lipoic acid, pantothenic acid, Fe, Cu, and Zn all have a role in heme metabolism, which regulates the release of reactive oxidants [31].

There are a few limitations to the study. Although using an MM supplement has practical applications because it is representative of a regular human diet, which is made up of a large number of vitamins and other compounds with antioxidant properties, using an MM supplement negates the possibility of identifying the precise compound or compounds responsible for overcoming the effects of oxidative stress. Another issue with these supplements is dosage; for instance, there have been reports that the RDA levels of vitamins C and E are insufficient to avoid oxidative damage. On the contrary, a lot of customers consume large amounts of antioxidant supplements, which may result in pro-oxidant effects [32]. Therefore, it is necessary to assess an individual's oxidative stress level prior to providing the supplement therapy. However, there are no defined reference values for an individual's oxidative stress status, and measuring oxidative stress is challenging and expensive.

## Conclusions

The findings of this investigation reinforced the idea of free radical participation as a physiological reaction to stress as well as the protective effect of MM via an antioxidant pathway. Therefore, MM can be recommended as a comprehensive supplement for health improvement and is anticipated to offer considerable benefits, especially to individuals with unbalanced diets, such as the elderly, children, the obese, and the underprivileged. Furthermore, MM supplementation can benefit nutritionally challenged individuals suffering from life-threatening illnesses such as cancer, cardiovascular disease, and other pathological conditions, since they experience significant levels of psychological and physical stress that might undermine their therapy. Further studies are warranted to explore the role of specific nutrients and their interactions in an MM supplement, which will aid in the development of more effective supplements.
